# Severe persistent mycobacteria antigen stimulation causes lymphopenia through impairing hematopoiesis

**DOI:** 10.3389/fcimb.2023.1079774

**Published:** 2023-01-18

**Authors:** Fei Li, Yanlin Ma, Xiaoping Li, Dan Zhang, Jiangyuan Han, Daquan Tan, Youjun Mi, Xiaojuan Yang, Juan Wang, Bingdong Zhu

**Affiliations:** ^1^ Gansu Provincial Key Laboratory of Evidence-Based Medicine and Clinical Translation and Lanzhou Center for Tuberculosis Research, Institute of Pathogen Biology, School of Basic Medical Sciences, Lanzhou University, Lanzhou, China; ^2^ Inpatient Ward 1, Lanzhou Pulmonary Hospital, Lanzhou, China; ^3^ Institute of Pathophysiology, School of Basic Medical Sciences, Lanzhou University, Lanzhou, China; ^4^ Department of Clinical Medicine, Gansu University of Traditional Chinese Medicine, Lanzhou, China; ^5^ State Key Laboratory of Veterinary Etiological Biology, College of Veterinary Medicine, Lanzhou University, Lanzhou, China

**Keywords:** mycobacterium tuberculosis, miliary tuberculosis, BCG, hematopoiesis, lymphopenia, IL-7

## Abstract

Miliary tubersculosis (TB), an acute systemic blood disseminated tuberculosis mainly caused by *Mycobacterium tuberculosis* (*M. tuberculosis*), can cause signs of lymphopenia in clinical patients. To investigate whether/how persistent mycobacteria antigen stimulation impairs hematopoiesis and the therapeutic effect of interleukin-7 (IL-7), a mouse model of Mycobacterium Bovis Bacillus Calmette-Guérin (BCG) intravenous infection with/without an additional stimulation with *M. tuberculosis* multi-antigen cocktail containing ESAT6-CFP10 (EC) and Mtb10.4-HspX (MH) was established. Consistent with what happened in miliary TB, high dose of BCG intravenous infection with/without additional antigen stimulation caused lymphopenia in peripheral blood. In which, the levels of cytokines IFN-γ and TNF-α in serum increased, and consequently the expression levels of transcription factors *Batf2* and *IRF8* involved in myeloid differentiation were up-regulated, while the expression levels of transcription factors *GATA2* and *NOTCH1* involved in lymphoid commitment were down-regulated, and the proliferating activity of bone marrow (BM) lineage^-^ c-Kit^+^ (LK) cells decreased. Furthermore, recombinant Adeno-Associated Virus 2-mediated IL-7 (rAAV2-IL-7) treatment could significantly promote the elevation of BM lymphoid progenitors. It suggests that persistent mycobacteria antigen stimulation impaired lymphopoiesis of BM hematopoiesis, which could be restored by complement of IL-7.

## 1 Introduction

Tuberculosis (TB) is one of the most serious infectious diseases worldwide (Harding, 2020). Miliary TB caused by hematogenous dissemination of *Mycobacterium tuberculosis* (*M. tuberculosis*) is a type of severe tuberculosis (Kim et al., 1990), which shows symptoms of systemic bacteremia, and has an acute-onset dangerous condition with many complications such as anemia and lymphopenia (Cucin et al., 1973; Achi et al., 2013). The current acknowledgment of lymphopenia during *M. tuberculosis* infection mainly focuses on the changes in peripheral T cells, including inhibiting T-cell proliferation and inducing lymphocyte apoptosis. It is well-known that *M. tuberculosis* could activate macrophages, which selectively inhibit T-cell proliferation through cell-cell contact and soluble factors derived from culture supernatants (Holt, 1979; Grange, 1998). *M. tuberculosis* antigen could also induce apoptosis in T lymphocytes (Soruri et al., 2002). However, recent studies suggest that *M. tuberculosis* infection or persistent antigen stimulation could impair hematopoiesis (Kaufmann et al., 2018; Li et al., 2019; Khan et al., 2020).

The hematopoietic system is the foundation of an immune response, in which hematopoietic stem cells (HSCs) could generate multipotent progenitors (MPP), which further differentiate into common lymphoid progenitors (CLP) and common myeloid progenitors (CMP). CLP is the key progenitor of mature lymphocytes (Kondo et al., 2003). HSCs and progenitor cells do not express any lineage-restricted marker, but highly express c-Kit (CD117) (Di Scala et al., 2015). CLP has roughly defined as Lineage^−^ Sca-1^int^ CD127^+^ c-Kit^int^ cells (Dakic et al., 2005). CMP can differentiate into megakaryocyte/erythrocyte progenitors (MEP) and granulocyte/macrophage progenitors (GMP), which further generate non-lymphoid leukocytes including neutrophils and monocytes (Paul et al., 2016). Intravenously immunized Mycobacterium Bovis Bacillus Calmette-Guérin (BCG) could get access to BM and change the transcriptional landscape of HSCs and MPP, driving HSC differentiation toward myeloid cells (Kaufmann et al., 2018; Cirovic et al., 2020). These studies highlighted the crucial roles of myelopoiesis in generating protective innate immunity against tuberculosis. However, chronic infection with *Mycobacterium avium (M. avium)* (Matatall et al., 2016) and persistent *M. tuberculosis* antigen stimulation impaired lymphoid commitment due to persistent IFN-γ stimulation and induced immune exhaustion (Li et al., 2019).

Hematopoietic lineage commitment is instructed by some inflammatory cytokines. Among them, IFN-γ impaired the self-renewal of HSCs and promoted myeloid differentiation (Matatall et al., 2016). Our previous study found that persistent *M. tuberculosis* antigen stimulation induced high levels of IFN-γ and impaired lymphopoiesis of BM hematopoiesis (Li et al., 2019). We also found that the inflammatory cytokine IFN-γ in the serum of IL-7 knockout mice was significantly increased (**Supplementary Figure 1**), which was similar to the cytokine profile in the animal model of this study. IL-7 is critical for human T cell lymphopoiesis, involved in lymphocyte generation and maintenance of T cell homeostasis (Fry and Mackall, 2005). Infection might impair the function of mesenchymal progenitor cells, which decreases the potential of secreting IL-7, further causing damage to CLPs (Terashima et al., 2016). In addition, high levels of IFN-γ could cause an increase of SOCS in pre-B stage cells and inhibit IL-7 signaling (Corfe et al., 2011). Thus, we hypothesized that supplementation of IL-7 might be a possible way to restore abnormal hematopoiesis.

Patients with miliary TB have signs of lymphopenia, but the mechanism is still unclear. In this study, the peripheral blood cell count extracted from hospital records in miliary TB patients was analyzed. Then BCG bloodstream infection model was applied to mimic the immunopathological changes of miliary TB infection, and the changes in hematopoiesis and immune system were studied. Furthermore, to investigate whether IL-7 could help reverse impaired hematopoiesis, the mice were treated with recombinant Adeno-Associated Virus 2-mediated IL-7 (rAAV2-IL-7) and the therapeutic effects were analyzed. By combining clinical case data with animal experiments, the pathogenesis of lymphopenia in miliary TB was revealed from the perspectives of the development and differentiation of BM hematopoietic cells, which provided a reference for the prevention and treatment of miliary TB.

## 2 Materials and methods

### 2.1 Patient’s enrollment and data collection

To analyze the changes in peripheral blood cell counts in patients with miliary TB, 28,047 cases of tuberculosis from Lanzhou Pulmonary Hospital from 2015 to 2019, including 104 cases of miliary TB, were enrolled. All of the individuals enrolled in this study were miliary TB patients, who were first hospitalized adults (18-81 years old), before anti-tuberculosis drug treatment and diagnosed by clinical presentation, medical history and comorbidities, chest computed tomography (CT) images, Chest X-ray (CXR) examination and laboratory results such as sputum smear, culture. Blood samples were collected before the first week of anti-tuberculosis treatment. The peripheral blood cell count data extracted from the electronic medical records were investigated in this study.

### 2.2 Constructing animal models of BCG infection with/without antigen stimulation

Specific pathogen-free C57BL/6 female mice (6-8 weeks old) (Gansu University of Traditional Medicine, Gansu, China) were infected with BCG (Shanghai strain, D2-PB302, a derivative of Copenhagen strain, supplied by Lanzhou Institute of Biological Products) at a dose of 5 × 10^7^ bacterial colony forming units (CFU) *via* intravenous injection (high BCG *i.v*. infection group) or intranasal route (high BCG *i.n*. infection group). The mice were also infected with a low dose of BCG (5 × 10^5^ CFU/ml) *via* the intranasal route (low BCG *i.n*. infection group), or injected with PBS as the sham control (PBS group). The peripheral blood cell count, cytokines production, the proliferation, and transcriptional differentiation of BM hematopoietic Lineage^-^ c-Kit^+^ cells (LK) were detected respectively at 3 weeks, 5 weeks, and 8 weeks after infection.

Furthermore, to mimic the process of continuous antigen stimulation during miliary TB, another mouse model of high-dose BCG plus *M. tuberculosis* antigen stimulation was established. The C57BL/6 mice were infected with BCG at a dose of 5 × 10^7^ CFU/ml *via* intravenous injection, followed by subcutaneous injection with *M. tuberculosis* antigen-based fusion protein ESAT6-CFP10 (10 μg) and Mtb10.4-HspX (MH) (Niu et al., 2011) (10 μg) weekly for four weeks. The mice injected with PBS were used as the sham control. At 5 weeks after infection, the peripheral blood cell count, the cytokine secretion, and the percentage of lymphoid progenitors in BM were detected.

### 2.3 The treatment of rAAV2-IL-7

The construction and quantification of rAAV2-IL-7 are performed as described previously (Han et al., 2021). In this study, the mice from the high BCG plus Ag group were treated with rAAV2-IL-7 *via* intraperitoneal injection at a dose of 5 × 10^8^ PFU/100 µl once one week after BCG infection (rAAV2-IL-7 treated group). The mice treated with rAAV2-EGFP were used as the sham control. The peripheral blood cell count, the levels of IL-7, IFN-γ, and TNF-α in serum, and the percentage of lymphoid progenitors in BM were detected at 5 weeks after BCG infection.

### 2.4 The peripheral blood cell count

The fresh blood from mice (about 100 μl) was taken into the anti-coagulation tube, which was filled with anticoagulant K3 EDTA. Then, the blood cells count from different groups was measured by an animal blood analyzer (Hemavet 950FS multispecies haematology analyzer, Drew Scientific, Oxford, UK).

### 2.5 Detecting cytokine profile in serum

The mice were infected intravenously with BCG at a dose of 5 × 10^7^ CFU. At 1, 2, 3, and 4 weeks later, the fresh blood (500 μl) was respectively collected. After blood centrifugation, the sera were collected to detect the cytokines profile including IL-2, IL-4, IL-5, IL-6, IL-9, IL-10, IL-13, IL-17A, IL-17F, IL-22, IFN-γ, TNF-α, IL-1α, IL-1β, IL-3, IL-7, IL-11, IL-12p40, IL-12p70, IL-23, IL-27, IL-33, IFN-β, GM-CSF and TSLP using LEGEND plex™ multi-analyte flow assay kit (Mouse Th Cytokine Panel 13-plex (Cat 740740) and Mouse Cytokine Panel 2 (Cat 740134), BioLegend, United States). The results of flow cytometry were converted to pg/mL production by BioLegend LEGENDplex software.

After rAAV2-IL-7 treatment, the sera of mice were also collected to detect the levels of IL-7, IFN-γ, and TNF-α by ELISA according to the manufacturer’s protocols (Mouse IL-7 ELISA Kit, AssayGenie, America; Mouse IFN-γ/TNF-α ELISA Kit, Dakewe Biotech Co., Ltd, Shenzhen, China).

### 2.6 LK cells isolation and flow cytometry analysis for the proliferating activity of LK cells

Following BCG infection for 3 weeks, 5 weeks, and 8 weeks, whole BM cells, isolated from femurs and tibias of mice, were passed through 30 µm pre-Separation Filters (#130-041-407, Miltenyi Biotech, Germany) to remove cell clumps. Then, the c-Kit positive cells were enriched by microbeads conjugated to monoclonal anti-mouse c-Kit antibodies (#130-091-224, Miltenyi Biotech, Germany). Subsequently, the lineage^-^ cells among them were obtained according to the manufacturer’s protocol (Mouse lineage cell depletion kit, #130-090-858, Miltenyi Biotech, Germany). The lineage-committed cells were magnetically enriched by the quadroMACS Starting Kit. The remaining cells were Lineage^-^ c-Kit^+^ cells (LK cells).

To mimic the pathological process that mycobacteria could reach the BM and affect cytokine production, thereby changing the BM microenvironment during chronic tuberculosis infection, LK cells (5 × 10^6^ cells/well) were stimulated *in vitro* with PPD (5 μg/ml) and cultured in RPMI 1640 supplemented with 10% FBS in 6-well plates. EdU (Click-iT™ EdU Flow Cytometry Assay Kit, Invitrogen, OR, USA) was incubated with LK cells at a final concentration of 30 μM at 37°C for 3 days. Then, these cells were collected by PBS, fixed, permeabilized, and incubated with Click-iT reaction buffer according to the Click-iT™ EdU Flow Cytometry Assay Kit’s protocol (Yu et al., 2009). Subsequently, these cells were stained with anti-mouse monoclonal antibodies, including APC-conjugated anti-Lineage (BD Pharmingen) 1μl and PE-conjugated anti-c-Kit (CD117, 2B8, eBioscience) 1μl in the dark at 4°C for 30 min. Finally, 10^5^ cells were analyzed by NovoCyte flow cytometer (ACEA, USA).

### 2.7 Analysis of transcription factor expression by quantitative RT-PCR

After LK cells were magnetically enriched, RNA was extracted using the TRIzol Reagent (Invitrogen, Grand Island, NY, USA) and reversely transcribed to cDNA (20 µl) according to the PrimeScript™ RT Kit’s instructions (TaKaRa, Dalian, China). The expression levels of transcription factors *GAPDH*, *Batf2*, *IRF8*, *GATA2*, and *NOTCH1* were detected by qRT-PCR, which was performed on the Step One Plus™ Real-Time PCR System (Applied Biosystems, ABI, USA) and the primers used were the same as our previews study (Li et al., 2019).

### 2.8 Flow cytometry analysis for lymphoid progenitors

At 5 weeks after BCG infection, whole BM cells were isolated from femurs and tibias, and subsequently passed through 30 µm pre-Separation Filters (#130-041-407, Miltenyi Biotech, Germany) to remove cell clumps. Then, the cells were resuspended were stained with APC-conjugated anti-Lineage (BD Pharmingen) 20 μl, PE-conjugated anti-c-Kit (CD117, 2B8, eBioscience) 1 μl and PerCP-Cyanine 5.5-conjugated anti-IL-7Ra (CD127, A7R34, Invitrogen) 1 μl in the dark at 4°C for 40 min. Finally, 10^6^ cells were analyzed by NovoCyte flow cytometer (ACEA, USA). Lineage^-^ c-Kit^+^ CD127^+^ cells were roughly defined as lymphoid progenitors.

### 2.9 Statistical methods

All values are expressed as mean ± standard error of the mean (SEM). The statistical analysis of clinical data was performed with an unpaired *t*-test to reduce bias. Differences between the variance were analyzed by one-way ANOVA or *t*-test using GraphPad Prism 5/8 software (GraphPad Software, USA). A value of *p*<0.05 was considered significant, and values of *p*<0.01 were considered statistically significant.

## 3 Results

### 3.1 Hematological abnormalities in miliary TB patients

Complete blood count data, extracted from the medical record at the time of patient admission, were analyzed. A total of 104 adult miliary TB patients (53 men and 51 women) were included in this study: miliary TB patients with lymphopenia (n = 80) and without lymphopenia (n = 24). The results showed that hematological abnormalities in miliary TB were as follows: leukopenia, defined as the absolute leukocyte count < 4 × 10^9^/L, was presented in 24 of 104 (23.08%) patients; lymphopenia, defined as lymphocyte percentage (the percentage of lymphocyte count to leukocyte count) < 20%, was common and was seen in 80 of 104 (76.92%) patients; 55.77% patients showed decreased lymphocyte count (< 1.5 × 10^9^/L). In addition, neutrophilia, defined as neutrophil percentage (the percentage of neutrophil count to leukocyte count) > 70%, was presented in 74 of 104 (71.15%) patients. It was found that the absolute lymphocyte count and lymphocyte percentage in miliary TB with lymphopenia group was lower than that in miliary TB without lymphopenia group (*p*<0.0001), while the absolute neutrophil count and neutrophil percentage in miliary TB with lymphopenia group was higher than that in miliary TB without lymphopenia group (*p*<0.05), suggesting that miliary TB patients were often accompanied by lymphopenia. There were no differences in the absolute leukocyte count, absolute monocyte count, hemoglobin, absolute erythrocyte count, and platelet count between miliary TB with lymphopenia and without lymphopenia groups. In addition, the common comorbidities of miliary TB were hypertension and diabetes (**Table 1**). The data demonstrated that hematological manifestations, especially lymphopenia and neutrophilia, commonly happened in miliary TB.

**Table 1:** Haematological findings of miliary TB.

Characteristics	Total (n = 104)	Patients with abnormal values		TB with lymphopenia (n = 80)	TB without lymphopenia (n = 24)	*P* value
Age (years)	39.7 ± 17.24	–	–	39.6 ± 18.19	40.3 ± 13.57	0.8599†
Male + Female	53 + 51	–	–	45 + 35	8 + 16	–
Absolute leukocyte count (× 10^9^/L)	5.6 ± 2.34	> 10	4 (3.85%)	5.9 ± 2.51	4.9 ± 1.46	0.0928†
		< 4	24 (23.08%)			
Absolute lymphocyte count (× 10^9^/L)	0.8 ± 0.47	> 4	0	0.6 ± 0.3	1.3 ± 0.52	**< 0.0001†**
		< 0.8	58 (55.77%)			
Lymphocyte percentage (%)	14.5 ± 8.87	> 40	3 (2.88%)	10.9 ± 4.00	27.0 ± 9.71	**< 0.0001†**
		< 20	80 (76.92%)			
Absolute neutrophil count (× 10^9^/L)	4.3 ± 2.13	> 7	9 (8.65%)	4.7 ± 2.22	3.1 ± 1.10	**0.0011†**
		< 2	7 (6.73%)			
Neutrophil percentage (%)	75.9 ± 14.24	> 70	74 (71.15%)	80.1 ± 12.55	61.8 ± 9.87	**< 0.0001†**
		< 50	3 (2.88%)			
Absolute monocyte count (× 10^9^/L)	0.5 ± 0.30	> 0.45	47 (45.19%)	0.5 ± 0.31	0.6 ± 0.24	0.2016†
		–	–			
Monocyte percentage (%)	8.8 ± 4.43	> 10	36 (34.62%)	8.1 ± 4.30	11.2 ± 3.98	**0.0028†**
		< 3	5 (4.81%)			
Hemoglobin (g/L)	119.9 ± 19.52	> 160 male, 140 female	3 (2.88%)	117.9 ± 18.96	126.5 ± 19.87	0.0565†
		< 120 male, 110 female	39 (37.50%)			
Absolute erythrocyte count (× 10^12^/L)	4.3 ± 0.66	> 5.5	3 (2.88%)	4.2 ± 0.67	4.4 ± 0.62	0.3078†
		< 3.5	11 (10.58%)			
Platelet count (× 10^9^/L)	198.1 ± 78.65	> 300	11 (10.58%)	190.8 ± 81.83	221.7 ± 61.81	0.0951†
		< 100	10 (9.62%)			
Comorbidities						
Hypertension	6 (5.8%)	–	–	5 (6.3%)	1 (4.2%)	–
Diabetes	3 (2.9%)	–	–	3 (3.8%)	0	–

† The results between miliary TB with lymphopenia and without lymphopenia groups were analyzed with an unpaired t-test. The data were shown as mean ± SEM, and p-value < 0.05 was considered a significant difference and was shown in bold text.

Lymphocyte percentage, the percentage of lymphocyte count to leukocyte count; Neutrophil percentage, the percentage of neutrophil count to leukocyte count; Monocyte percentage, the percentage of monocyte count to leukocyte count.

### 3.2 High-dose BCG bloodstream infection causes lymphopenia in mice

Following BCG infection, the changes in peripheral blood cell count in mice were analyzed. Five weeks after BCG infection, the counts of leukocytes, lymphocytes, and platelets in high BCG *i.v.* infection group was significantly lower than that in PBS group (*p*<0.05) compared with PBS group, although the counts of neutrophils in high BCG *i.v.* infection group did not change significantly. However, high BCG *i.v.* infection group showed an increased neutrophil percentage than that in PBS group and low BCG *i.n.* infection group, while the lymphocyte percentage was reduced (*p*<0.05) (**Table 2**). These results indicated that high-dose BCG infection could induce lymphopenia and high neutrophil percentage, which is consistent with that in miliary TB patients.

**Table 2:** Haematological changes at 5 weeks after BCG infection in mice.

Groups	RBC (× 10^12^/L)	LY (× 10^9^/L)	NEUT (× 10^9^/L)	MO (× 10^9^/L)	LEU (× 10^9^/L)	LY percentage (%)	NEUT percentage (%)	MO percentage (%)
PBS group	10.15 ± 0.453	8.45 ± 1.16	1.68 ± 0.210	0.35 ± 0.030	10.50 ± 1.16	80.28 ± 2.78	16.17 ± 2.88	3.31 ± 0.25
Low BCG *i.n*. group	11.24 ± 0.022 ^a^	7.25 ± 1.08	1.84 ± 0.144	0.21 ± 0.067 ^a^	9.31 ± 0.99	77.49 ± 3.32	20.19 ± 3.52	2.21 ± 0.60 ^a^
High BCG *i.n.* group	8.71 ± 0.598 ^ab^	5.29 ± 2.94	2.18 ± 0.585	0.24 ± 0.044 ^a^	7.76 ± 2.36	61.51 ± 21.97	34.34 ± 20.37	3.01 ± 0.97
High BCG *i.v.* group	9.464 ± 0.634 ^bc^	4.25 ± 1.05 ^ab^	1.97 ± 0.290	0.23 ± 0.052 ^a^	6.53 ± 1.27 ^ab^	64.57 ± 4.92 ^ab^	30.67 ± 3.88 ^ab^	3.59 ± 0.71 ^b^

RBC, erythrocyte; LY, lymphocyte; NEUT, neutrophil; MO, monocyte; LEU, leukocyte; LY percentage, the percentage of lymphocyte count to leukocyte count; NEUT percentage, the percentage of neutrophil count to leukocyte count; MO percentage, the percentage of monocyte count to leukocyte count. ^a^ p < 0.05, compared with PBS group; ^b^ p < 0.05, compared with Low BCG i.n. group; ^c^ p < 0.05, compared with the High BCG i.n. group. n = 5-8.

Eight weeks after BCG infection, the counts of leukocytes, lymphocytes, and platelets in high BCG *i.v.* infection group was significantly lower than that in other groups (*p*<0.05). Compared with PBS group and high BCG *i.n.* infection group, the neutrophil counts in high BCG *i.v.* infection group was also lower (*p*<0.05). However, the erythrocyte counts and the percentages of lymphocytes and neutrophils did not change significantly between groups (**Table 3**). These results indicated that high-dose BCG bloodstream infection caused lymphopenia.

**Table 3:** Haematological changes at 8 weeks after BCG infection in mice.

Groups	RBC (× 10^12^/L)	LY (× 10^9^/L)	NEUT (× 10^9^/L)	MO (× 10^9^/L)	LEU (× 10^9^/L)	LY percentage (%)	NEUT percentage (%)	MO percentage (%)
PBS group	10.46 ± 0.98	7.27 ± 0.74	2.10 ± 0.35	0.20 ± 0.08	9.69 ± 0.91	75.06 ± 3.94	21.66 ± 3.14	2.06 ± 0.78
Low BCG *i.n.* group	10.51 ± 0.78	7.95 ± 1.48	2.08 ± 0.65	0.25 ± 0.09	10.46 ± 2.38	76.84 ± 3.56	19.48 ± 2.16	2.35 ± 0.39
High BCG *i.n.* group	10.40 ± 0.49	8.46 ± 2.19	2.33 ± 0.71	0.33 ± 0.11	11.22 ± 2.90	75.64 ± 3.52	20.63 ± 3.07	2.88 ± 0.30
High BCG *i.v.* group	9.28 ± 0.79	4.70 ± 0.34 ^abc^	1.19 ± 0.12 ^ac^	0.22 ± 0.08	6.14 ± 0.46 ^abc^	76.59 ± 1.02	19.49 ± 1.58	3.57 ± 1.29

a p < 0.05, compared with PBS group; bp < 0.05, compared with Low BCG i.n. group; cp < 0.05, compared with the High BCG i.n. group. n = 5-8.

### 3.3 High-dose BCG bloodstream infection induced high levels of IFN-γ and TNF-α

**Figure 1:**
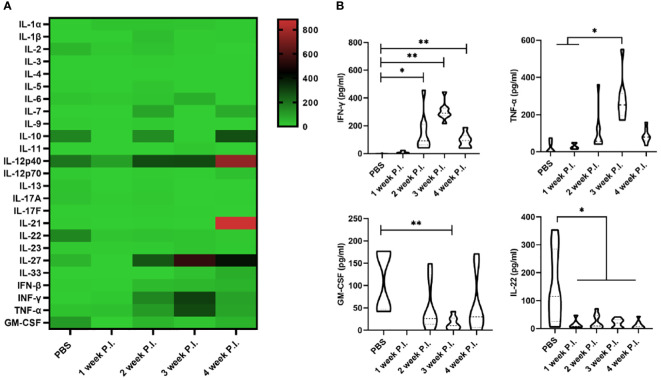
The change in cytokine profile induced by high-dose BCG bloodstream infection. Mice were infected with BCG *via* intravenous injection, at 1, 2, 3, and 4 weeks after infection, the variation of serum cytokine profile was detected by LEGEND plex™ Multi-Analyte Flow Assay Kit. **(A)** Heatmap showing highly secreted cytokines (red) and low secreted cytokines (green). **(B)** The levels of cytokines (including IFN-γ, TNF-α, GM-CSF, and IL-22) that has changed significantly. P.I., post-infection. *n* = 3-4, * *p* < 0.05, ** *p* < 0.01.

To investigate the changes in cytokine profile induced by BCG infection, we evaluated the serum cytokine profile. The results showed that at 3 weeks after BCG infection, the levels of IFN-γ and TNF-α induced by high BCG *i.v*. infection was significantly higher than those in PBS group (*p*<0.01), while the levels of GM-CSF and IL-22 were lower compared with those in PBS group. Of note, the increase of IFN-γ and TNF-α secretion was most obvious at 3 weeks after BCG infection, while IFN-γ levels gradually decreased at 4 weeks compared with the levels at 3 weeks in high BCG *i.v*. infection group. No significant changes could be observed in other levels of detected cytokines (**Figure 1**).

### 3.4 High-dose BCG bloodstream infection reduced the proliferating activity of LK cells in BM

**Figure 2:**
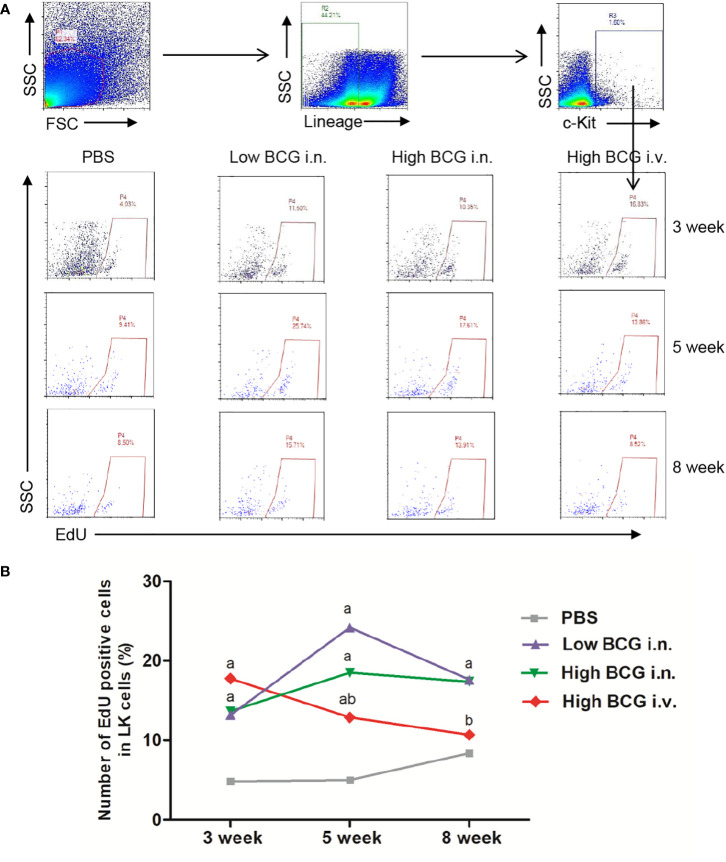
High-dose BCG bloodstream infection reduced the proliferating activity of LK cells. Mice were infected with BCG *via* intravenous injection or intranasal route. At 3 weeks, 5 weeks, and 8 weeks after infection, BM cells were isolated and the proliferating activity of LK cells was determined by flow cytometry. **(A)** Gating strategy for detecting EdU incorporation into LK cells in BM. **(B)** Number of EdU-positive cells in LK cells. ^a^ *p* < 0.05, compared with PBS group; ^b^ *p* < 0.05, compared with Low BCG *i.n*. group; *n* = 5.

To investigate whether the hematopoiesis was impaired, we detected the proliferating activity of hematopoietic cells. Following BCG infection, the proliferating activity of LK cells in BM was analyzed by determining the EdU-stained DNA in proliferating cells *in vitro*. The results showed that at 3 weeks after BCG infection, the proliferation rate of LK cells in high BCG *i.v*. infection group (17.76 ± 5.71%) was significantly higher than that in PBS group (4.85 ± 2.95%) (*p*<0.01). The differences between high BCG *i.v*. infection group and PBS group gradually decrease at 5 weeks and 8 weeks (**Figure 2**). These data demonstrated that high-dose BCG bloodstream infection reduced the proliferating activity of LK cells.

### 3.5 High-dose BCG bloodstream infection regulated the expression of transcription factors involved in the differentiation of LK cells

**Figure 3:**
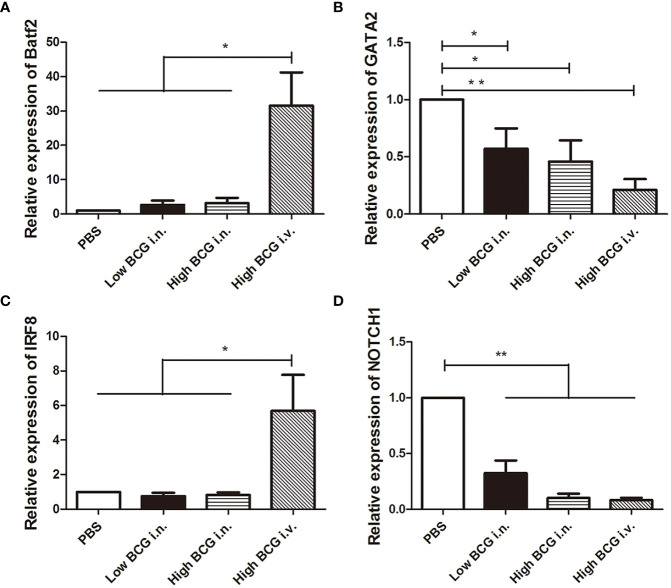
The expression of transcription factors involved in the differentiation of LK cells during high-dose BCG bloodstream infection. Mice were infected with BCG *via* intravenous injection or intranasal route. At 5 weeks after infection, LK cells were enriched with Lineage and c-Kit positive microbeads. Then, the relative quantities of transcription factors *Batf2*, *IRF8*, *GATA2*, and *NOTCH1* mRNA in LK cells were determined by qRT-PCR. *n* = 5, * *p* < 0.05, ** *p* < 0.01.

The differentiation of HSCs was closely regulated by lineage-determining transcription factors. Following BCG infection, the expression of transcription factors involved in the differentiation of LK cells was analyzed. Four key transcription factors are chosen for analysis: Batf2 and IRF8 (involved in myeloid differentiation) and GATA2 and NOTCH1 (governing lymphoid commitment) (Doulatov et al., 2012; Matatall et al., 2016). The results showed that the expression levels of transcription factors in LK cells from high BCG *i.v.* infection group changed significantly compared with PBS group: the expression levels of *Batf2* and *IRF8* were markedly up-regulated, while the expression levels of *GATA2* and *NOTCH1* were significantly down-regulated (**Figure 3**). It indicated that high-dose BCG bloodstream infection might promote myeloid differentiation and repress lymphoid commitment.

### 3.6 rAAV2-IL-7 treatment partially restored lymphopenia induced by high-dose BCG plus *M. tuberculosis* antigen stimulation

To mimic the process of continuous antigen stimulation during miliary TB, mice were infected with BCG and boosted with *M. tuberculosis* antigens weekly for four weeks. Five weeks after infection, the changes in peripheral blood cell count were analyzed. The results showed that the lymphocyte percentage in BCG plus Ag group (60.06 ± 4.58%) was lower than that in PBS group (77.49 ± 3.13%) (*p*<0.05). Moreover, high BCG plus Ag group also showed an increased neutrophil percentage than that in PBS group. All these data indicated that high-dose BCG infection plus *M. tuberculosis* antigen stimulation could induce lymphopenia, which is consistent with what happened in high BCG *i.v*. infection model and miliary TB patients (**Table 4**).

**Table 4:** Haematological changes after IL-7 treatment in mice.

Groups	RBC (× 10^12^/L)	LY (× 10^9^/L)	NEUT (× 10^9^/L)	MO (× 10^9^/L)	LEU (× 10^9^/L)	LY percentage (%)	NEUT percentage (%)	MO percentage (%)
PBS group	9.56 ± 0.77	4.43 ± 0.69	1.05 ± 0.25	0.20 ± 0.09	5.73 ± 0.90	77.49 ± 3.13	18.40 ± 2.85	3.36 ± 1.31
Untreated control	7.55 ± 1.77 ^a^	1.25 ± 0.32 ^a^	0.60 ± 0.10 ^a^	0.17 ± 0.07 ^a^	2.07 ± 0.43 ^a^	60.06 ± 4.58 ^a^	29.48 ± 3.87 ^a^	7.86 ± 1.97 ^a^
rAAV2-EGFP	8.64 ± 1.70 ^b^	0.92 ± 0.09 ^a^	0.73 ± 0.23 ^a^	0.18 ± 0.05 ^a^	1.85 ± 0.28 ^a^	49.98 ± 4.89 ^ab^	38.48 ± 6.87 ^ab^	9.79 ± 2.92 ^a^
rAAV2-IL-7 treated	10.29 ± 1.31 ^b^	1.97 ± 0.16 ^abc^	0.97 ± 0.07 ^b^	0.25 ± 0.09 ^a^	3.18 ± 0.19 ^abc^	60.28 ± 1.77 ^ac^	30.41 ± 1.61 ^ac^	7.87 ± 2.33 ^a^

a p < 0.05, compared with PBS group; bp < 0.05, compared with untreated control group; cp < 0.05, compared with rAAV2-EGFP group. n = 4-10.

Considering that persistent *M. tuberculosis* antigen stimulation induced high levels of IFN-γ, which impaired lymphopoiesis and inhibit IL-7 signaling, and IL-7 was critical for human T cell lymphopoiesis, we explored the intervention effect of IL-7 in this model. To explore whether IL-7 treatment could reverse lymphopenia, the mice were treated with rAAV2-IL-7 and the changes in peripheral blood cell count were analyzed. The rAAV2-IL-7 treated group showed an increased lymphocyte percentage (60.28 ± 1.77%) than rAAV2-EGFP group (49.98 ± 4.89%) (*p*<0.05). Moreover, the neutrophil percentage in rAAV2-IL-7 treated group (30.41 ± 1.61%) was lower than that in rAAV2-EGFP group (38.48 ± 6.87%) (*p*<0.05) (**Table 4**). All these results indicated that rAAV2-IL-7 treatment could partially restore the numbers of lymphocytes, which suggests that the abnormal hematopoiesis caused by high BCG plus Ag stimulation could be partially reversed by IL-7.

### 3.7 rAAV2-IL-7 supplementation contributed to maintaining the homeostasis of IFN-γ and TNF-α production in mice stimulated with high BCG plus *M. tuberculosis* antigen stimulation

To investigate whether high-dose BCG plus *M. tuberculosis* antigen stimulation induced the alteration in cytokine secretion, the levels of IL-7, IFN-γ, and TNF-α in serum were analyzed. The results showed that the IL-7 levels in high BCG plus Ag group (274.3 ± 32.40 pg/ml) were significantly lower than that in PBS group (324.4 ± 14.67 pg/ml) (*p*<0.01). In contrast, high BCG plus Ag group showed a higher level of IFN-γ (142.6 ± 5.85 pg/ml) than that in PBS group (16.7 ± 2.75 pg/ml). Meanwhile, the levels of TNF-α in high BCG plus Ag group (64.1 ± 20.72 pg/ml) were higher than that in PBS group (29.6 ± 6.73 pg/ml). These data indicated that high-dose BCG plus *M. tuberculosis* antigen stimulation reduced IL-7 secretion, and increased the production of IFN-γ and TNF-α.

**Figure 4:**
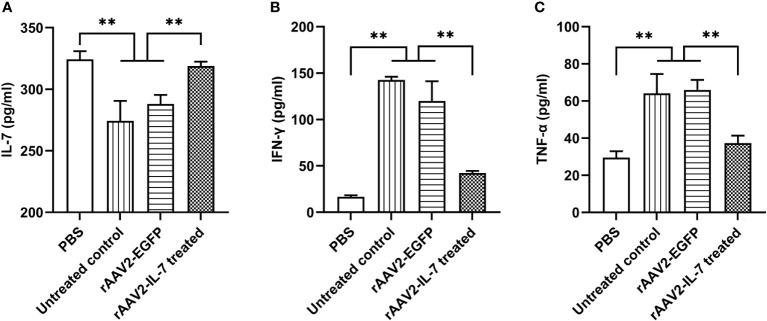
rAAV2-IL-7 treatment increased the IL-7 levels and decreased IFN-γ and TNF-α levels. The C57BL/6 mice from high BCG plus Ag group were treated with rAAV2-IL-7 or rAAV2-EGFP. At 5 weeks after infection, the IL-7, IFN-γ, and TNF-α levels in serum were detected. **(A)** The levels of IL-7. **(B)** The levels of IFN-γ. **(C)** The levels of TNF-α. *n* = 4-6, ** *p* < 0.01.

As expected, rAAV2-IL-7 treated group showed a higher level of IL-7 (318.8 ± 7.35 pg/ml) than that in untreated group (274.3 ± 32.40 pg/ml) and rAAV2-EGFP control group (288.1 ± 14.57 pg/ml) (**Figure 4A**). Besides, rAAV2-IL-7 treated group showed a lower level of IFN-γ (42.2 ± 4.0 pg/ml) in serum than untreated group (142.6 ± 5.85 pg/ml) and rAAV2-EGFP group (120.1 ± 36.6 pg/ml) (**Figure 4B**). Also, the levels of TNF-α in rAAV2-IL-7 treated group (37.3 ± 6.95 pg/ml) was lower than that in untreated group (64.1 ± 20.72 pg/ml) and rAAV2-EGFP group (66.0 ± 9.30 pg/ml) (*p*<0.01) (**Figure 4C**). All these data indicated that rAAV2-IL-7 supplementation contributed to maintaining the homeostasis of IFN-γ and TNF-α secretion in mice stimulated with high BCG plus *M. tuberculosis* antigen.

### 3.8 rAAV2-IL-7 treatment partially increased the proportion of lymphoid progenitors caused by BCG plus *M. tuberculosis* antigen stimulation

**Figure 5:**
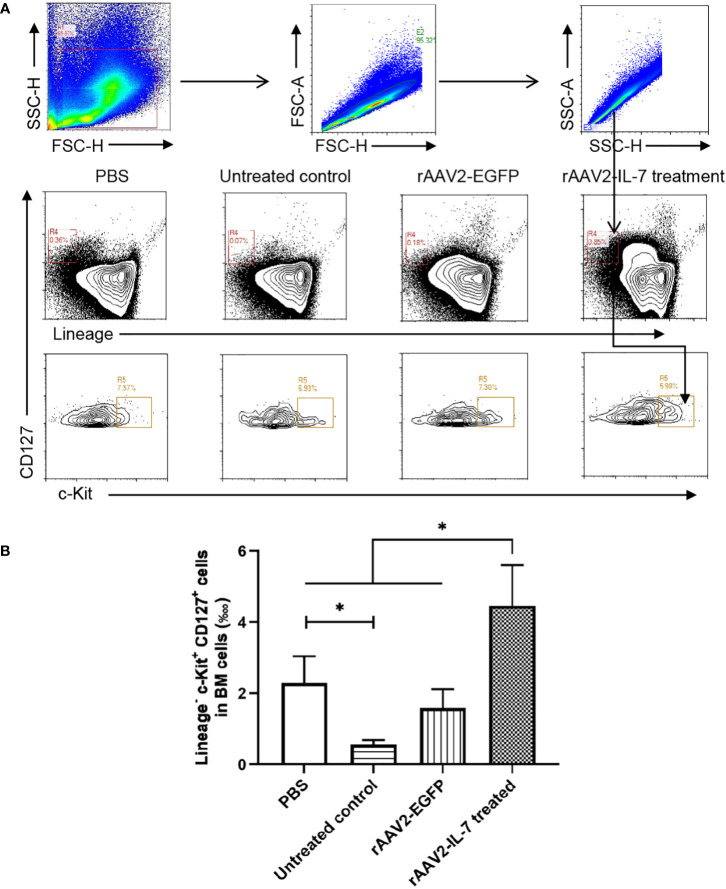
rAAV2-IL-7 treatment restored the reduced lymphoid progenitors caused by high-dose BCG plus *M. tuberculosis* antigen stimulation. At 5 weeks after infection, BM cells were isolated and the percentage of Lineage^-^ c-Kit^+^ CD127^+^ cells was determined by flow cytometry. **(A)** Gating strategy for detecting lymphoid progenitors. **(B)** The percentage of lymphoid progenitors. *n* = 5-6, * *p* < 0.05.

Following high-dose BCG plus *M. tuberculosis* antigen stimulation, the proportion of lymphoid progenitors in BM was analyzed. The results showed that the percentage of lymphoid progenitors (0.56 ± 0.11 ‱) was reduced compared with PBS group (2.29 ± 0.68 ‱). Following rAAV2-IL-7 treatment, the percentage of lymphoid progenitors (4.46 ± 1.04 ‱) was significantly higher than that in untreated group and rAAV2-EGFP group (1.58 ± 0.48 ‱) (*p*<0.05), even higher than PBS group (*p*<0.05) (**Figure 5**). Collectively, these results demonstrated that IL-7 treatment could restore the impaired hematopoiesis caused by high-dose BCG plus *M. tuberculosis* antigen stimulation.

## 4 Discussion

Our research and other reports found that lymphopenia often happened in miliary TB patients. Considering the possible damage to BM hematopoiesis, we established a mouse model of high-dose BCG intravenous infection with/without additional *M. tuberculosis* antigen stimulation, and detected the changes in cytokine secretion and BM hematopoiesis in it. We found that persistent mycobacteria antigen stimulation induced excessive expression of IFN-γ and TNF-α, which promoted myeloid differentiation, repressed lymphoid commitment, and reduced the proliferation activity of LK cells, ultimately leading to peripheral lymphopenia. Moreover, we investigated the therapeutic effects of IL-7 on it and found that rAAV2-IL-7 treatment could restore the proportion of BM lymphoid progenitors and peripheral blood lymphocyte count along with a decrease in the production of IFN-γ and TNF-α.

Lymphopenia was caused by multiple mechanisms, which may act together and overlap in some cases during miliary TB infection. First, *M. tuberculosis* infection inhibits T-cell proliferation and induces lymphocyte apoptosis (Holt, 1979; Grange, 1998; Soruri et al., 2002). Second, *M. tuberculosis* infection could induce lymphocyte traffic to the secondary lymphoid tissue (Goetzl and Gräler, 2004), et al. Furthermore, our findings suggest that *M. tuberculosis* affected the differentiation of BM precursor cells, resulting in decreased lymphocyte production.

To investigate the pathogenic mechanism of lymphopenia caused by miliary TB, we used a mouse model of BCG bloodstream infection to mimic the immunopathological changes of clinical miliary TB infection. The route and dose of BCG have a great impact on hematopoiesis. We used different routes and doses of BCG to infect mice and found that the changes in hematopoiesis varied among different groups. Low-dose BCG respiratory tract infection and high-dose BCG respiratory tract infection did not cause abnormal hematopoiesis, while high-dose BCG intravenously infection did. Our results showed that high-dose BCG bloodstream infection impaired BM hematopoiesis and caused lymphopenia, which was consistent with what happened in miliary TB. Our experiments indicated that high-dose BCG bloodstream infection could induce the production of inflammatory factors to mimic immunopathological changes in clinical miliary TB to some degree. BCG can elicit immune responses similar to natural infection (Zhou and Xie, 2014). BCG could be performed in Biosafety Level 2 (BSL-2) laboratory, which is convenient for us to study the mechanism of its pathological damage, overcome the biological safety and high cost of direct *M. tuberculosis* infection in mice, and help preliminary explore the effect of *M. tuberculosis* infection on hematopoiesis. However, BCG has deleted some virulence factors and cannot survive as long as *M. tuberculosis*. To overcome the limitations of BCG infection, the mice were infected with BCG plus *M. tuberculosis* antigen stimulation to mimic the immunopathological damage caused by continuous *M. tuberculosis* antigen stimulation, and explore the effect of mycobacterial infection on BM hematopoiesis. The results showed that high-dose BCG plus antigen stimulation also impaired cytokine secretion and BM hematopoiesis.

The route and dose of BCG administration and research focus may influence the effect of BCG immunization (Foster et al., 2021). Recent studies reported that intravenous BCG (5 × 10^7^ CFU) vaccination, rather than intradermal and aerosol BCG, shows strong protection against *M. tuberculosis* infection in highly susceptible rhesus macaques (Darrah et al., 2020). Another clinical trial found that BCG revaccination (intradermal BCG (2- 8 × 10^5^ CFU administration)) significantly improves the BCG-specific CD4 T cell responses, contributing to the prevention of *M. tuberculosis* infection in adolescents (Nemes et al., 2018). However, when converting the BCG dose in rhesus macaques or humans to mice, the dose was significantly lower. And the dose used in our experiment (5 × 10^7^ CFU) was far greater than the protective dose. Previously, compared with subcutaneous BCG administration, intravenous BCG (1 × 10^6^ CFU) vaccination could induce trained immunity through educating HSCs to generate macrophages, which confer enhanced protection against pulmonary *M. tuberculosis* in mice (Kaufmann et al., 2018). Both these studies and our study found that BCG immunization resulted in alterations in BM hematopoiesis and promoted myeloid differentiation. These studies highlighted the protective effect of BCG on tuberculosis by promoting myeloid differentiation and generating protective innate immunity, while our research focused on the side effect of impaired lymphoid commitment.

The homeostasis and differentiation of HSCs were regulated by some inflammatory cytokines (Petit et al., 2002; Hajishengallis et al., 2021). Pathogen infections could induce the proliferation and differentiation of HSPCs to increase the output of mature immune cells (Sato et al., 2009; Matatall et al., 2016). However, persistent IFN-γ stimulation could impair the proliferation and reconstitution of HSCs, and enhance the formation of monocyte at the expense of the production of neutrophils, erythrocytes, and B cells (de Bruin et al., 2014; Pascutti et al., 2016). IFN-γ promotes myeloid differentiation by upregulating the expression of *Batf2* during chronic *M. avium* infection (Matatall et al., 2016). Our studies also found that persistent *M. tuberculosis* antigen stimulation induced the persistent production of IFN-γ, which decreased the proliferating activity of hematopoietic cells, promoted myeloid differentiation, and inhibited lymphoid commitment (Li et al., 2019). Similar to IFN-γ, TNF-α also could inhibit HSCs proliferation (Jacobsen et al., 1992; Baldridge et al., 2011). GM-CSF is a hematopoietic cytokine that regulates the differentiation and maturation of granulocyte and macrophage lineages (Metcalf, 1989). IL-22, a member of the IL-10 family, together with GM-CSF were higher in PBS group than those in other groups, but their mechanisms remained to be explored.

IL-7 is crucial for controlling lymphoid homeostasis (Fry et al., 2001). In the BM microenvironment, IL-7 is mainly secreted by osteoblasts (Cordeiro Gomes et al., 2016). Sepsis-induced osteoblast ablation decreased the ability to secrete IL-7, thereby causing impaired lymphopoiesis (Terashima et al., 2016). In addition, high levels of IFN-γ could cause an increase of SOCS in pre-B stage cells and inhibit IL-7 signaling (Corfe et al., 2011). IL-7 plays a critical role in the survival or proliferation of CLPs. IL-7 could induce the expression of Bcl-2 and activate STAT5 to promote the survival of CLPs (Hofmeister et al., 1999; Goetz et al., 2005). IL-7 knockout mice showed a significant reduction in Sca-1^+^ CLPs cells (Tsapogas et al., 2011). Moreover, the mice with impaired IL-7 signaling showed a decrease in the number of early thymic progenitors, while the mice with overexpressing IL-7 greatly increased the numbers of early thymic progenitors (Plumb et al., 2017). Our studies also found that persistent antigen stimulation caused lower IL-7 levels in serum and decreased the proportion of lymphoid progenitors, while rAAV2-IL-7 treatment significantly promoted the elevation of BM lymphoid progenitors and partially reverse lymphopenia. In addition, IL-7 plays a critical role in T cell homeostasis (Fry et al., 2001), which is required for normal thymopoiesis (Murray et al., 1989), reduces apoptosis and promotes T cell survival (Vassena et al., 2007; Chen et al., 2021). IL-7 is also critical for promoting Treg development (Vang et al., 2008) and reducing IFN-γ, ultimately reducing the effect of elevated IFN-γ on hematopoietic function.

Altogether, IL-7 may maintain T cell homeostasis by acting on both peripheral T cells and BM hematopoiesis. A more extensive investigation on all possible mechanisms affecting peripheral blood cell count needs to be conducted.

## 5 Conclusion

Severe mycobacteria antigen stimulation induced persistent production of IFN-γ and TNF-α, which may impact hematopoiesis and lead to lymphopenia. IL-7 could reverse impaired hematopoiesis and lymphopenia by decreasing IFN-γ and TNF-α levels.

## Data availability statement

The original contributions presented in the study are included in the article/**Supplementary Material**. Further inquiries can be directed to the corresponding author.

## Ethics statement

The animal study was reviewed and approved by Institutional Animal Care and Use Committee of Lanzhou University (No. Jcyxy 20210402). Written informed consent was obtained from the individual(s) for the publication of any potentially identifiable images or data included in this article.

## Author contributions

FL, YMa, XL, DZ, JH, DT, YMi, XY, and JW performed experiments. FL, XL, and BZ designed experiments. FL, YMa, and BZ wrote and revised the manuscript. All authors contributed to the article and approved the submitted version.
