# Supplementary material for: Severe persistent mycobacteria antigen stimulation causes lymphopenia through impairing hematopoiesis
Source: Front Cell Infect Microbiol. 2023 Jan 18;13:1079774. doi: 10.3389/fcimb.2023.1079774 (PMC9889370; doi:10.3389/fcimb.2023.1079774)
Supplement: Supplementary file 1 [file DataSheet_1.docx]

Supplementary Material

# Graphical Abstract


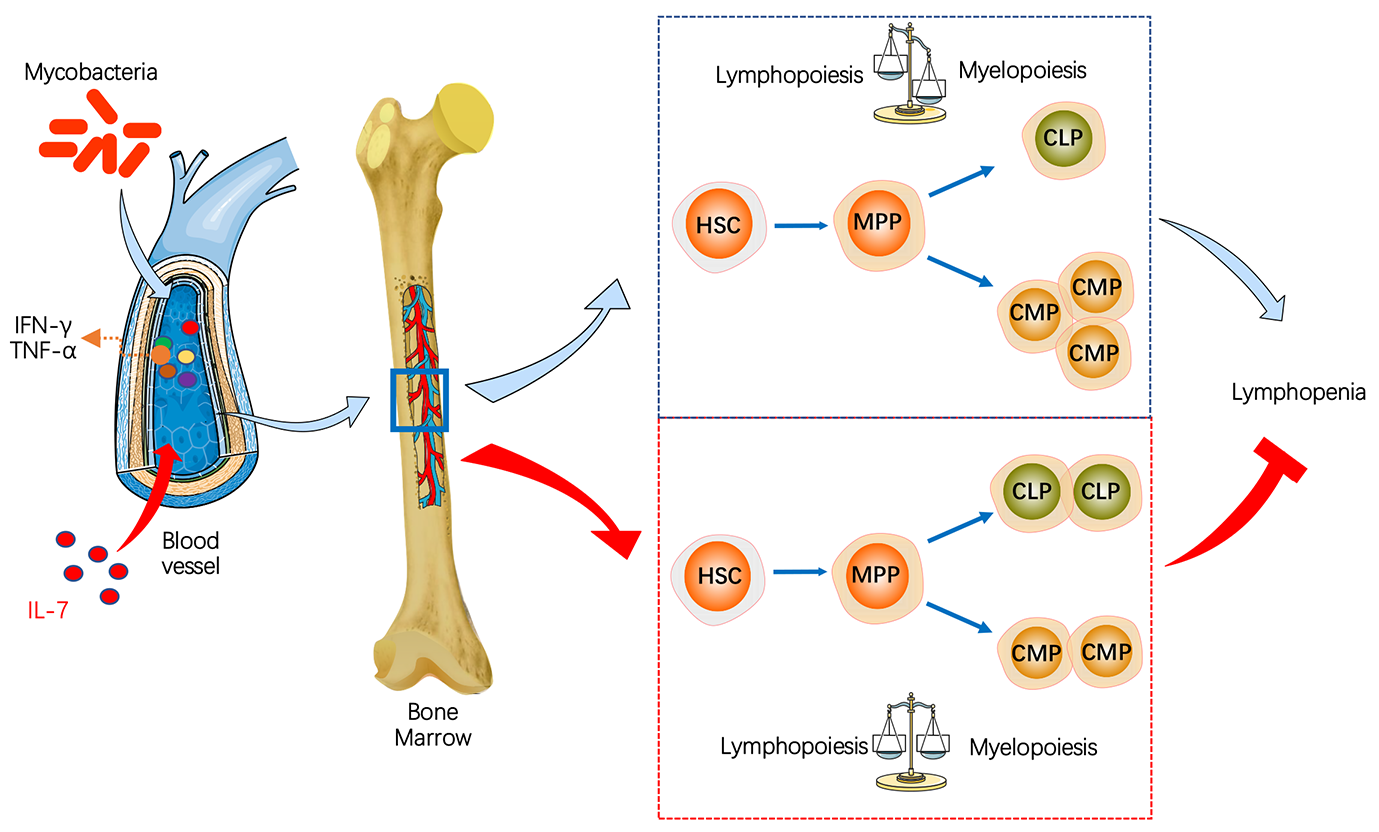


**Highlights**

Hematogenously disseminated mycobacteria could increase the production of IFN-γ and TNF-α.

Elevated IFN-γ and TNF-α may impact hematopoiesis and lead to lymphopenia.

IL-7 could reverse impaired hematopoiesis and lymphopenia caused by elevated IFN-γ and TNF-α during high-dose mycobacteria infection.

# Supplementary Figure


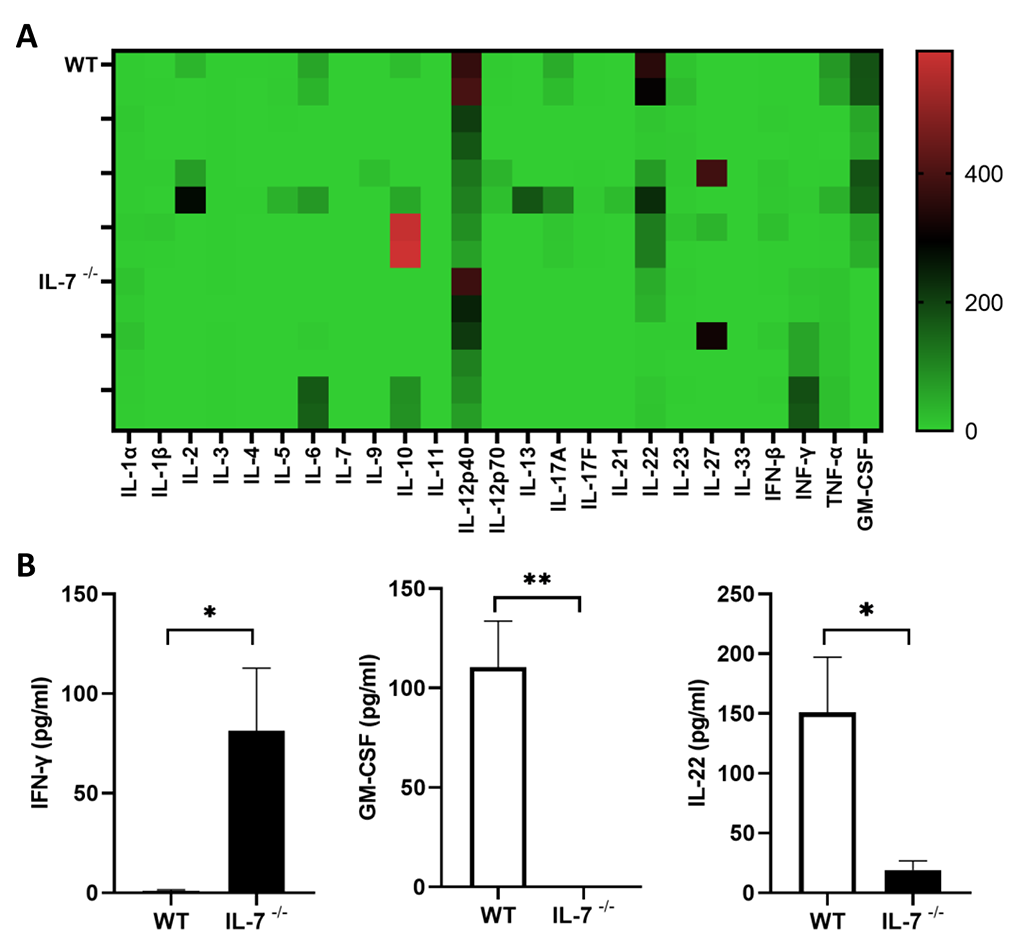


**Supplementary Figure 1.** The serum cytokine profile in IL-7^-/-^ mice. The sera from IL-7^-/-^ mice were taken to detect the variation of serum cytokine profile by LEGEND plex™ Multi-Analyte Flow Assay Kit. (A) Heat-map showing highly secreted cytokines (red) and low secreted cytokines (green). (B) The levels of cytokines (including IFN-γ, GM-CSF and IL-22) that has changed significantly. *n* = 3-4, * *p* < 0.05, ** *p* < 0.01.
